# Endotoxin Mediated-iNOS Induction Causes Insulin Resistance via ONOO^−^ Induced Tyrosine Nitration of IRS-1 in Skeletal Muscle

**DOI:** 10.1371/journal.pone.0015912

**Published:** 2010-12-28

**Authors:** Geneviève Pilon, Alexandre Charbonneau, Phillip J. White, Patrice Dallaire, Mylène Perreault, Sonia Kapur, André Marette

**Affiliations:** 1 Department of Medicine, Québec Heart and Lung Institute (Laval Hospital), Ste-Foy, Québec, Canada; 2 Laval University Hospital Research Center, Metabolism, Vascular and Renal Health Axis, Ste-Foy, Québec, Canada; Pennington Biomedical Research Center, United States of America

## Abstract

**Background:**

It is believed that the endotoxin lipopolysaccharide (LPS) is implicated in the metabolic perturbations associated with both sepsis and obesity (metabolic endotoxemia). Here we examined the role of inducible nitric oxide synthase (iNOS) in skeletal muscle insulin resistance using LPS challenge in rats and mice as *in vivo* models of endotoxemia.

**Methodology/Principal Findings:**

Pharmacological (aminoguanidine) and genetic strategies (iNOS^−/−^ mice) were used to counter iNOS induction *in vivo*. *In vitro* studies using peroxynitrite (ONOO^−^) or inhibitors of the iNOS pathway, 1400 W and EGCG were conducted in L6 myocytes to determine the mechanism by which iNOS mediates LPS-dependent insulin resistance. *In vivo*, both pharmacological and genetic invalidation of iNOS prevented LPS-induced muscle insulin resistance. Inhibition of iNOS also prevented insulin resistance in myocytes exposed to cytokine/LPS while exposure of myocytes to ONOO^−^ fully reproduced the inhibitory effect of cytokine/LPS on both insulin-stimulated glucose uptake and PI3K activity. Importantly, LPS treatment *in vivo* and iNOS induction and ONOO^−^ treatment *in vitro* promoted tyrosine nitration of IRS-1 and reduced insulin-dependent tyrosine phosphorylation.

**Conclusions/Significance:**

Our work demonstrates that iNOS-mediated tyrosine nitration of IRS-1 is a key mechanism of skeletal muscle insulin resistance in endotoxemia, and presents nitrosative modification of insulin signaling proteins as a novel therapeutic target for combating muscle insulin resistance in inflammatory settings.

## Introduction

Nitrosative modification of target molecules is becoming increasingly recognized as an important intracellular signaling mechanism. Rapidly reversible and regulated in large part by local nitric oxide (NO) status, this class of post-translational modification appears to be a selective process that targets specific cysteine and tyrosine residues to activate or restrict cellular events [1], [2], [3]. Unsurprisingly, accumulating evidence suggests that these modifications carry important implications for whole body physiology and are highly relevant to the pathophysiology of a growing number of disease states, particularly those with an inflammatory base.

Cellular NO availability is regulated by the activities of three nitric oxide synthase (NOS) isozymes. Two constitutively expressed Ca^2+^-dependent enzymes, the endothelial form (eNOS) and the neuronal form (nNOS), and a transcriptionally regulated inducible form (iNOS)[4], [5]. In contrast to the two constitutive members of the NOS family, iNOS, once induced, is capable of generating large amounts of NO for a prolonged period of time [6], [7], [8]. In fact, iNOS may produce up to one thousand times more NO than both eNOS and nNOS combined. As a result of this superior capacity, factors that regulate iNOS induction may exert a critical influence on cellular physiology via widespread induction or reduction of nitrosative modifications.

We first proposed that insulin resistance (IR) is linked to iNOS induction in skeletal muscle and other insulin target cells during systemic inflammation. We showed that administration of the endotoxin lipopolysaccharide (LPS), a model of acute systemic inflammation, induces iNOS in muscle, liver, and adipose tissue [9], [10]. Cytokine-mediated iNOS expression in muscle elevates basal glucose uptake but causes marked IR [9], [10], [11] and both can be abrogated by iNOS inhibition in muscle cells in vitro [11]. We further provided genetic evidence that iNOS induction in skeletal muscle modulates whole-body glucose metabolism and mediates skeletal muscle IR in obese high-fat fed mice [12]. This finding was in line with well documented evidence showing that obesity is a chronic inflammatory disorder characterized by increased expression of multiple inflammatory cytokines known to induce iNOS such as TNF-α, interferon-γ, IL-6, and IL-1β [13]. However, it is still unclear whether iNOS induction in acute models of systemic inflammation also underlies skeletal muscle IR. One study showed that mice lacking iNOS were protected from LPS-mediated IR as determined by an insulin tolerance test but skeletal muscle insulin sensitivity was not determined in this study [14]. Furthermore, despite numerous reports of elevated iNOS expression in both acute and chronic inflammatory settings the underlying molecular mechanism by which iNOS mediates skeletal muscle IR remains ill-defined. This question is also relevant to obesity-linked inflammation since the finding that LPS levels are increased in obese insulin resistant mice as a result of perturbations of the gut microbiota that cause metabolic endotoxemia [15].

Targeted protein S-nitrosylation is one form of nitrosative modification that has been suggested to contribute to the insulin desensitizing actions of iNOS [14], [16], [17]. The presence of nitrosothiol adducts on cysteine residues in glycosylated hemoglobin is elevated in patients with type 2 diabetes and it appears that in skeletal muscle of obese diabetic mice this form of nitrosative modification may be directly targeted towards key enzymes of the insulin signaling cascade. Indeed, increased S-nitrosylation of insulin signaling proteins has been reported in both obese and LPS-treated animals [14], [16], [17]. Interestingly, the presence of reactive oxygen species (ROS) was shown to enhance the impact of the NO donor SNAP on Akt activity [18]. Although this was apparently associated with enhanced S-nitrosylation of Akt, the potential for other complementary nitroxyradical mediated nitrosative modification such as tyrosine nitration of Akt was not determined in this study.

Indeed, NO is also known to readily react with superoxide (O_2_^−^) to form peroxynitrite (ONOO^−^) [19]. This powerful oxidant has been reported to modulate cellular functions through the nitration of tyrosine residues. The biological reactivity of ONOO^−^ for tyrosine nitration actually depends on radical intermediates arising from the interaction of ONOO^−^ with CO_2_ [20], [21]. The CO_3_^−^ radicals produced in this reaction generate secondary radicals on tyrosine residues with which the less reactive NO_2_ couples to form the nitrated product [21]. It is noteworthy that nitrated proteins have been detected in at least 50 human disorders such as Alzheimer disease, rheumatoid arthritis, glaucoma, asthma and inflammatory bowel disease [22], and multiple groups have reported that protein tyrosine nitration is elevated in plasma and tissues of type 2 diabetics [23], [24]. Moreover, it has been shown that nitration of tyrosine residues decreases their subsequent phosphorylation capacity [25]. We have recently reported that lipid infusion in mice causes hepatic IR by promoting iNOS induction and tyrosine nitration of insulin signaling proteins in liver [26]. Mice lacking iNOS were protected from this effect indicating that the iNOS/NO/ONOO^−^ pathway is a key mediator of hepatic IR upon lipid challenge. Whether tyrosine nitration also mediates iNOS-linked IR in skeletal muscle remains to be investigated. The aim of this study was therefore to determine the role of iNOS and ONOO^−^ in skeletal muscle IR using *in vivo* and *in vitro* models of endotoxemia, where iNOS induction is known to exert a pathological role.

## Results

### Inhibition of iNOS prevents IR in skeletal muscle of LPS-treated rats

**Figure 1:**
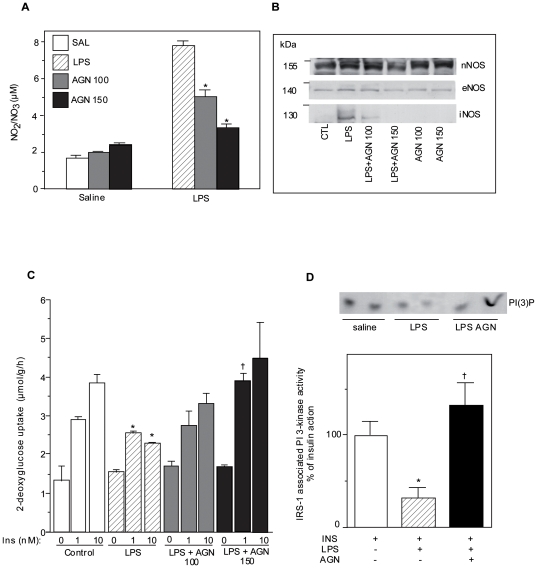
Effect of aminoguanidine on iNOS induced insulin resistance in muscle in vivo. Rats were injected i.p with LPS 20 mg/kg with or without aminoguanidine (100 or 150 mg/kg) and sacrified 6 hours later. A) Nitrite accumulation in plasma was measured by fluorometry. Data are mean ±SEM; *P<0.05 vs LPS control. B) NOS enzyme protein expression was measured by western blot in soleus muscle. C) Effect of LPS with or without aminoguanidine was measured on basal or insulin stimulated glucose transport in strips of soleus muscle. Data are mean ±SEM; *P<0.05 vs control, †P<0.05 vs LPS. D) IRS-1 associated PI 3-kinase activity was measured in muscle of LPS +/− aminoguanidine (150 mg/kg) treated animals. Insulin (3,8 U/kg i.v) was injected 4 minutes prior to sacrifice. Data are mean ±SEM; *P<0.05 vs insulin control, †P<0.05 vs LPS.

To study the role of iNOS in muscle IR, we treated rats with LPS in the presence or absence of the iNOS inhibitor aminoguanidine. LPS was found to induce iNOS as reflected by iNOS protein expression and plasma NO production (Figure 1A and B). As expected, pretreatment with aminoguanidine (100 or 150 mg/kg) blocked NO production and iNOS protein expression in a dose dependent manner (Figure 1A and B). However, the two constitutive NOS enzymes, eNOS and nNOS, were not affected by aminoguanidine treatment.

We next investigated the influence of iNOS induction on insulin sensitivity *ex vivo* by measuring glucose uptake in isolated soleus muscle from these rats. Insulin-stimulated glucose uptake was reduced in LPS-treated animals compared to the control group (Figure1C). Aminoguanidine treatment restored insulin-stimulated glucose uptake in LPS treated animals in a dose-dependent manner such that the lower dose of aminoguanidine partially improved insulin sensitivity while the higher dose fully restored muscle insulin action (Figure 1C).

We had previously reported that iNOS induction causes IR at least in part through inhibition of IRS-1 associated PI 3-kinase [12], [27]. Therefore, we determined whether the activity of this enzyme was altered in LPS-treated rats. LPS treatment decreased the ability of insulin to stimulate PI 3-kinase activity while iNOS inhibition with aminoguanidine fully restored insulin's action on PI 3-kinase (Figure 1D).

### Targeted iNOS disruption in mice protects against LPS-induced IR

**Figure 2:**
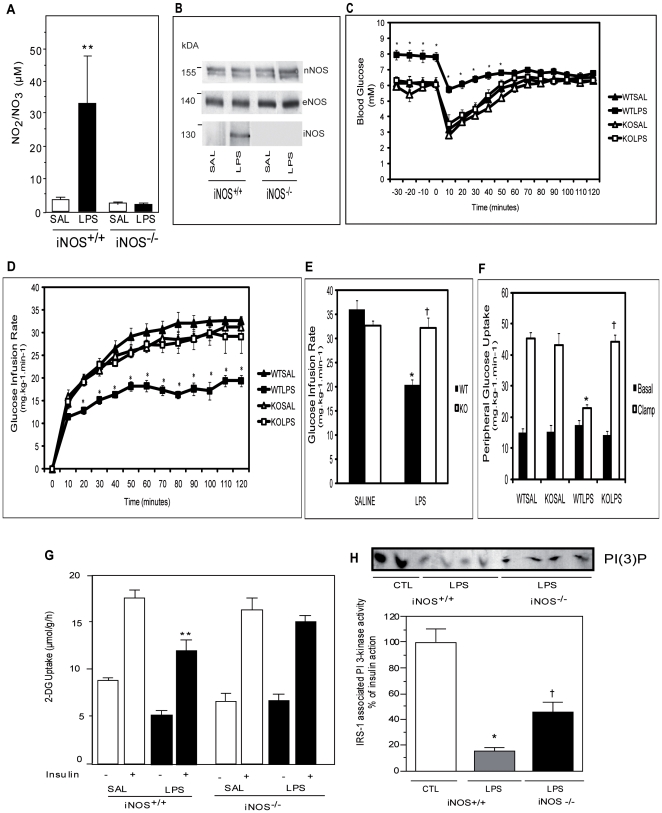
iNOS disruption protects mice against insulin resistance induced by LPS treatment. Wild type iNOS^+/+^(WT) or iNOS^−/−^ (KO) mice were treated with saline (SAL) or LPS A) nitrite accumulation in plasma was measured by fluorometry. Data are mean ±SEM; **P<0.01 vs saline control. B) representative blot of NOS Protein expression in skeletal muscle of type iNOS^+/+^ or iNOS^−/−^ mice treated with saline (SAL) or LPS. C) Blood glucose measured during the HIEC. Data are mean ±SEM; *P<0.05 vs iNOS^+/+^(WT) saline control. D) Glucose infusion rate during the HIEC. Data are mean ±SEM; *P<0.05 vs iNOS^+/+^(WT) saline control. E) Mean glucose infusion rate during the last 60 minutes of the HIEC. Data are mean ±SEM; *P<0.05 vs iNOS^+/+^(WT) saline control, †P<0.05 vs iNOS^+/+^(WT) LPS. F) Basal and Clamp peripheral glucose uptake. Data are mean ±SEM; *P<0.05 vs iNOS^+/+^(WT) saline control, †P<0.05 vs iNOS^+/+^(WT) LPS. G) Basal and insulin stimulated 2-Deoxy-glucose uptake in isolated soleus of iNOS^+/+^ or iNOS^−/−^ mice treated with saline (SAL) or LPS. Data are mean ±SEM; **P<0.01 vs iNOS^+/+^(WT) saline insulin control. H) Insulin induced IRS-1-associated PI 3-kinase activity. Data are mean ±SEM; *P<0.05 vs iNOS^+/+^ control (CTL), †P<0.05 vs iNOS^+/+^ LPS.

Since aminoguanidine can also inhibit the formation of advanced glycation endproducts [28] we next used a genetic model of iNOS disruption to further demonstrate the role of iNOS in LPS-induced IR. As expected, LPS challenge induced iNOS and subsequent NO production was observed in wild-type (iNOS^+/+^) mice but was fully abrogated in iNOS^−/−^ littermates (Figures 2A and B). As with aminoguanidine inhibition, genetic deletion of iNOS was associated with protection from LPS-induced IR. Indeed, as expected iNOS^+/+^ LPS treated mice displayed elevated basal glycemia (Figure 2C) and a reduced glucose infusion rate compared to saline treated controls (Figure 2D and E) during the hyperinsulinemic-euglycemic clamp (HIEC). This was not the case in LPS-treated iNOS^−/−^ mice whose basal glycemia and glucose infusion rate during the HIEC was similar to that of saline treated mice. In fact, saline treated iNOS^−/−^ mice did not vary from their iNOS^+/+^ counterparts for any of the parameters measured during the HIEC. Importantly, the LPS-mediated skeletal muscle IR revealed by reduced peripheral glucose uptake in iNOS^+/+^ mice was completed prevented in iNOS^−/−^ mice (Figure 2F). This finding was further supported by *ex vivo* glucose transport experiments in which insulin-mediated 2-deoxyglucose uptake was impaired in LPS-treated iNOS^+/+^ animals but normalized in the soleus of LPS-treated iNOS^−/−^ mice compared to their saline treated controls (Figure 2G). Moreover, LPS-mediated inhibition of insulin-stimulated phosphatidylinositol 3-kinase (PI3K) activity in skeletal muscle was also significantly improved by iNOS deletion (Figure 2H).

**Figure 3:**
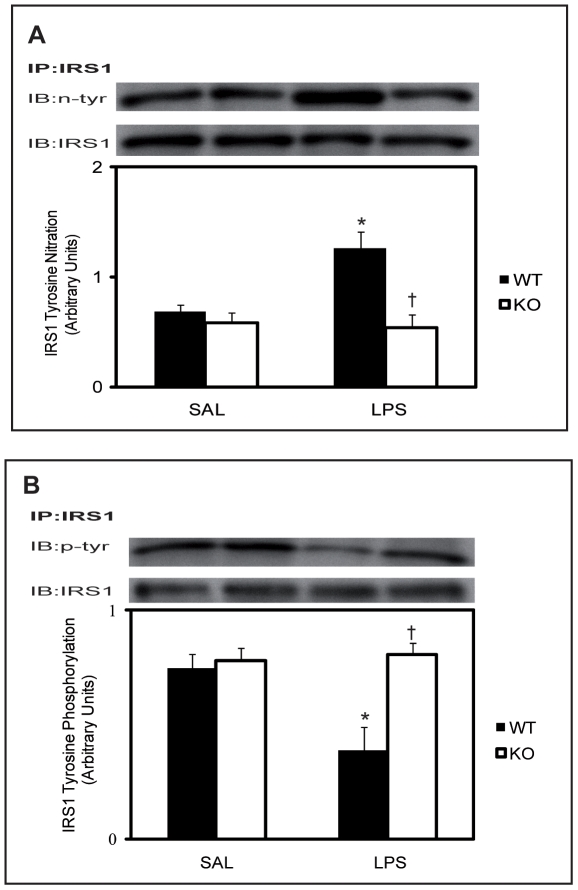
Effect of LPS treatment on tyrosine nitration and phosphoylation of IRS-1 in mouse skeletal muscle. A) Skeletal muscle lysates from wild type iNOS^+/+^(WT) or iNOS^−/−^ (KO) mice treated with saline (SAL) or LPS were immunoprecipitated with anti-IRS-1 antibody and then immunoblotted with anti-nitrotyrosine antibody B) lysates were also immunoprecipitated with anti-phosphotyrosine antibody (4G10) and then immunoblotted using the IRS-1 antibody. The quantification shown below the representative gels was normalized for total IRS-1. Data are mean ±SEM; *P<0.05 vs iNOS^+/+^ saline control (SAL), †P<0.05 vs iNOS^+/+^ LPS.

To gain further understanding of the molecular mechanisms underlying iNOS-induced IR we next examined IRS-1 tyrosine nitration and phosphorylation in skeletal muscle from saline and LPS-treated iNOS^+/+^ and iNOS^−/−^ mice that had undergone the HIEC procedure. We found a marked increase in tyrosine nitration of IRS-1 in the muscle of LPS-treated iNOS^+/+^ mice compared to their saline treated counterparts (Figure 3A). Remarkably tyrosine nitration of IRS-1 was completely abrogated in LPS-treated iNOS^−/−^ mice. Importantly the increase in tyrosine nitration of IRS-1 was also associated with a concomitant decrease in tyrosine phosphorylation *in vivo* (Figure 3B).

### iNOS inhibits insulin-stimulated glucose uptake via the production of ONOO^−^

**Figure 4:**
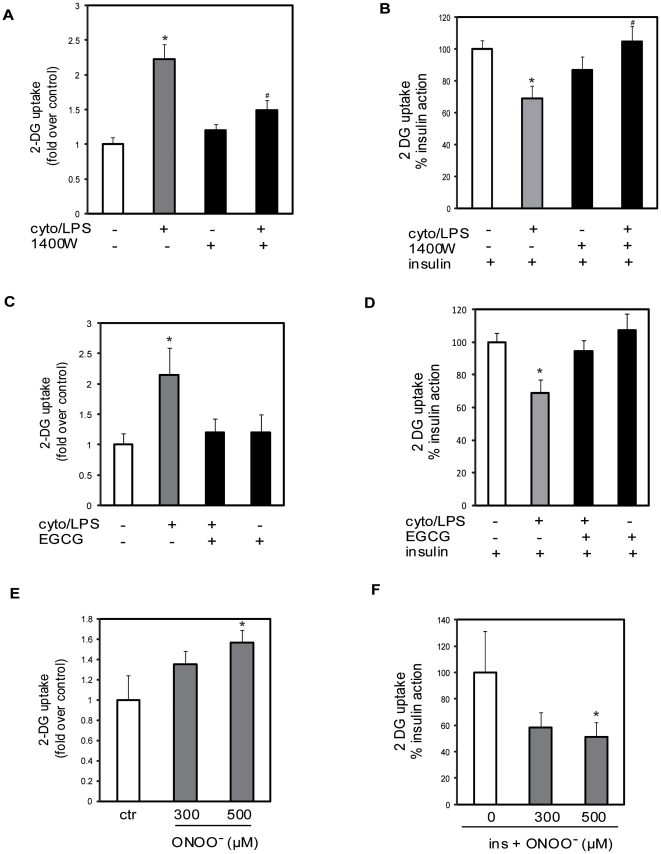
Effect of ONOO^−^ and iNOS induction on glucose transport in L6 muscle cells. The effect of cyto/LPS on basal (panel A) and insulin stimulated (panel B) glucose uptake was measured in L6 muscle cells treated with TNF-α (10 ng/ml), IFN-γ (200 units/ml) and LPS (10 µg/ml) for 24 hours, +/− 1400 W expressed as fold change or percent insulin action respectively. Data are mean ±SEM; *P<0.05 vs control, #P<0.05 vs cyto/LPS. (C) The effect of EGCG (10 µM) on basal glucose transport in the presence of cytokines/LPS. Data are mean ±SEM; *P<0.05 vs control. (D) The effect of EGCG(10 µM) on insulin stimulated glucose transport in the presence of cytokines/LPS. Data are mean ±SEM; *P<0.05 vs control. (E) Basal glucose transport in the presence of increasing concentrations of ONOO^−^. Data are mean ±SEM; *P<0.05 vs control (ctr). F) Insulin-stimulated glucose transport in the presence of increasing concentrations of ONOO^−^. Data are mean ±SEM; *P<0.05 vs control.

We next explored whether iNOS-mediated IR and IRS-1 tyrosine nitration could be recapitulated *in vitro* in muscle cells exposed to endotoxin and inflammatory cytokines. The cellular model also allows us to test whether ONOO^−^ may be involved in iNOS-linked tyrosine nitration of IRS-1 and inhibition of insulin signaling to PI3K. Exposure of muscle cells to cytokine/LPS for 24 hrs markedly induced NO production as detected by the accumulation of nitrite in the culture medium and this was completely inhibited by co-incubation with the iNOS inhibitor 1400 W (ctr: 0.03 +/− 0.07 µM, cyto/LPS: 9.18 +/− 0.62 µM, cyto/LPS+1400 W: not detected). In agreement with our previous observations that iNOS induction increases GLUT1 expression in L6 myocytes [11], cytokine/LPS treatment increased basal glucose transport (Figure 4A). This elevated basal glucose uptake was not observed in rat or mouse muscles likely because of the shorter time of LPS exposure (6 hrs) as compared to the 24 hrs treatment of muscle cells with the cytokine/LPS mixture. Importantly, co-incubation with the iNOS inhibitor 1400 W blunted the cytokine/LPS-mediated increase in basal glucose uptake. Cytokine/LPS exposure also significantly decreased insulin-stimulated glucose transport (Figure 4B) and this impairment was also reversed by iNOS inhibition. These results clearly support the *in vivo* findings showing a role for iNOS in LPS–induced IR.

We also studied the effect of epigallocathechin gallate (EGCG), an alternative inhibitor of the iNOS pathway, in cytokine/LPS treated skeletal myocytes [29]. As observed with 1400 W-mediated-iNOS inhibition, EGCG treatment also blunted the increase of basal glucose transport and reversed the deleterious effect of iNOS induction on insulin-stimulated glucose uptake (Figures 4C and 4D). Next, we treated cells directly with authentic ONOO^−^. As observed for cytokine/LPS mediated-iNOS induction, ONOO^−^ treatment stimulated basal glucose transport while inhibiting insulin-stimulated glucose uptake (Figures 4E and 4F). These data imply that the reactive nitrogen derivative ONOO^−^ may be responsible for the deleterious effects of iNOS on insulin-stimulated glucose transport.

### ONOO^−^ reproduces the inhibitory effect of iNOS induction on PI3K activity

**Figure 5:**
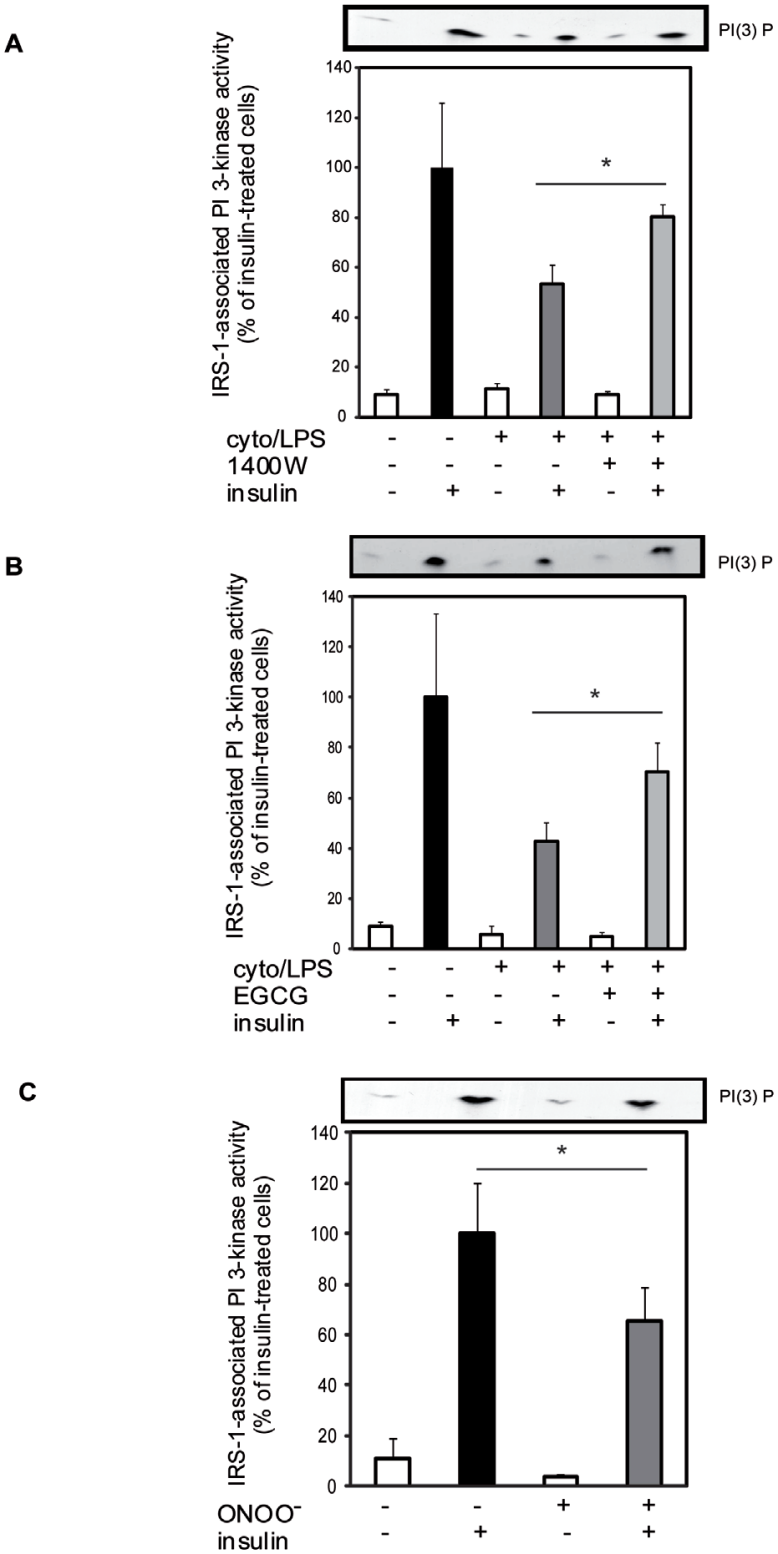
Effect of iNOS and ONOO^−^ on IRS-1-associated PI 3-kinase activity in L6 myocytes. PI 3-kinase was measured in anti-IRS-1 immunoprecipitates of L6 cells treated with A) cyto/LPS with TNF-α (10 ng/ml), IFN-γ (200 units/ml) and LPS (10 µg/ml) for 24 hours, +/− iNOS inhibitor 1400 W (0,1 mM), B) +/− EGCG (10 µM) or C) in cells directly treated for 1 hour with ONOO^−^ . Quantification of ^32^P incorporated into PI 3-phosphate (PI 3P) was expressed relative to insulin control value. A representative autoradiograph is shown at the top of the figure. Data are mean ±SEM; *P<0.05 vs the indicated group.

Reminiscent of the *in vivo* study, we observed an association between the cytokine/LPS-induced defect in insulin-stimulated glucose transport and impaired insulin signal transduction through IRS-1/PI3K in the cellular model. Cytokine/LPS treatment caused ∼50% reduction in insulin-stimulated IRS-1-associated PI3K activity as compared to control cells (Figures 5A and 5B). Importantly, both 1400 W and EGCG restored insulin-stimulated PI3K activity in cytokine/LPS treated cells, while exposure to authentic ONOO^−^ mimicked the cytokine/LPS treatment and blunted the ability of insulin to induce IRS-1 associated PI3K activity (Figure 5C). Neither 1400 W nor EGCG alone modulated PI3K activity (data not shown) and no effect of cytokine/LPS or ONOO^−^ was observed on basal PI3K activity. These results support a role for ONOO^−^ in the iNOS-induced defect in insulin-stimulated PI3K activity in skeletal muscle cells.

### ONOO^−^ causes tyrosine nitration of IRS-1

**Figure 6:**
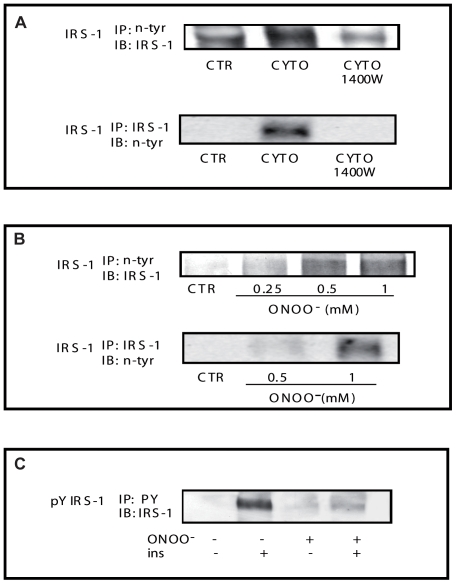
Effect of ONOO^−^ and iNOS induction on IRS-1 tyrosine nitration and phosphorylation. A) Protein lysates from cells treated with and cytokines +/− the iNOS inhibitor 1400 W (0.1 mM) were immunoprecipitated with anti-nitrotyrosine antibody and then immunoblotted with anti-IRS-1 antibody or immunoprecipitated with anti-IRS-1 antibody and then immunoblotted with anti-nitrotyrosine antibody. B) Protein lysates from cells treated with increasing doses of ONOO^−^ were immunoprecipitated with anti-nitrotyrosine antibody and then immunoblotted with anti-IRS-1 antibody or immunoprecipitated with anti-IRS-1 antibody and then immunoblotted with anti-nitrotyrosine antibody. C) L6 cells were serum deprived for 4 hours and then treated with or without ONOO^−^ for 1 hour. Cells were then stimulated with 100 nM insulin for the last 5 minutes of the treatment. Cell lysates were immunoprecipitated with anti-phosphotyrosine antibody (4G10) and then immunoblotted using the IRS-1 antibody. Gels are representative of two independent experiments.

Since free ONOO^−^ interacts with proteins to form nitrotyrosine adducts, we screened for the presence of this modification on IRS-1, a critical element for insulin action that was determined to be a site of defective insulin signaling in both the animal and cellular models studied here. To evaluate specifically the nitration of IRS-1 we first immunoprecipitated proteins containing nitrotyrosine adducts using the antinitrotyrosine antibody and probed for IRS-1 in lysates from L6 muscle cells treated with or without cytokine/LPS in the absence or presence of 1400 W. In line with our findings concerning insulin signaling to PI3K, cytokine/LPS treatment was found to promote tyrosine nitration of IRS-1 and this was blunted by treatment with the iNOS inhibitor 1400 W (Figure 6A). These results were confirmed by performing the reverse experiment where IRS-1 was first immunoprecipitated, followed by western blotting with the anti-nitrotyrosine antibody. Importantly, increasing doses of ONOO^−^ were also found to promote tyrosine nitration of IRS-1(Figure 6B).

We next determined whether nitration of IRS-1 on tyrosine residues could decrease its capacity for insulin-stimulated tyrosine phosphorylation. To do so we measured IRS-1 tyrosine phosphorylation in cells treated with insulin in the absence or presence of ONOO^−^. We found that ONOO^−^ treatment significantly decreased insulin-induced tyrosine phosphorylation on IRS-1 (Figure 6C). Taken together these data indicate that ONOO^−^ mediated tyrosine nitration of IRS-1 largely accounts for iNOS-induced IR in skeletal muscle cells.

## Discussion

The advent of metabolic endotoxemia as an important contributor to obesity-linked inflammation and IR has renewed scientific interest in defining the mechanisms underlying LPS-induced perturbations in glucose metabolism [15]. Herein, using both *in vivo* and *in vitro* models of endotoxemia we delineated the important role that iNOS-dependent tyrosine nitration of the proximal insulin signaling intermediate, IRS-1, plays in endotoxemia-induced skeletal muscle IR. Our data not only establish a key role for iNOS but also suggest a role for the reactive nitrogen derivative ONOO^−^ in the metabolic consequences of iNOS induction in skeletal muscle. Indeed, exposure to authentic ONOO^−^ was able to fully reproduce the effect of cytokine/LPS-mediated iNOS induction on glucose transport, insulin signaling to PI3K, IRS-1 tyrosine nitration and phosphorylation. Importantly, the use of iNOS^−/−^ mice allowed us to provide genetic evidence for iNOS-dependent IRS-1 tyrosine nitration in skeletal muscle *in vivo*.

Until now our understanding of the molecular mechanisms by which iNOS mediates IR in skeletal muscle was limited to NO mediated S-nitrosylation [18]. In the present work we show for the first time that the radical NO oxidation product ONOO^−^ also contributes to iNOS-mediated insulin signaling defects in skeletal muscle via tyrosine nitration of IRS-1. This post-translational modification appears to impede insulin signal transduction by blocking tyrosine phosphorylation of IRS-1 and thereby limiting its interaction with PI3K.

Our report of a negative role for elevated tyrosine nitration in skeletal muscle cells exposed to cytokines and LPS or authentic ONOO^−^ is in line with observations made in other cell types. To date ONOO^−^ mediated tyrosine nitration has been shown to negatively modulate the function of several enzymes such as glutamine synthase, prostacyclin synthase, cytochrome P450, tyrosine hydrolase and sarcoplasmic reticulum Ca-ATPase [30], [31], [32], [33], [34]. Furthermore, tyrosine nitration has also been associated with impaired PI 3-kinase activity in endothelial cells, macrophages and 3T3-L1 adipocytes exposed to authentic ONOO^−^ *in vitro* [30], [31], [32], [33], [34].

Although studies in cells had previously shown the potential for authentic ONOO^−^ to impair insulin stimulated PI 3-kinase activity *in vitro* it was not yet known whether this was a physiologically relevant occurrence in major insulin target tissues. However, we recently reported that iNOS dependent tyrosine nitration of IRS-1 and IRS-2 underlies lipid-induced IR in liver [26]. In this study, we found that acute 6 h lipid infusion like LPS treatment promotes iNOS induction in liver and this is accompanied by IR and tyrosine nitration of insulin signaling intermediates that is completely prevented in liver of iNOS^−/−^ mice. In the present paper we now provide evidence that iNOS-linked IRS-1 tyrosine nitration also underlies skeletal muscle IR in the acute inflammatory setting of endotoxemia. Furthermore this *in vivo* finding was recapitulated in L6 myocytes where we show that chronic cytokine/LPS treatment or acute exposure to authentic ONOO^−^ increased tyrosine nitration of IRS-1, resulting in decreased IRS-1 tyrosine phosphorylation and impaired activation of PI3K, leading to defective insulin stimulation of glucose transport. These effects were reversed by pre-treatments with inhibitors of the iNOS pathways (1400 W and EGCG). Moreover, both cytokine/LPS and ONOO^−^ treatments were found to promote tyrosine nitration of IRS-1. Again, these defects were corrected by iNOS inhibition with 1400 W, further supporting the concept that IRS-1 tyrosine nitration by ONOO^−^ underlies iNOS-induced muscle IR. Importantly, as with most other post-translational modifications, tyrosine nitration is a selective process and not all tyrosine residues present in proteins are nitrated. To date no specific consensus sequence or motif has been identified for protein tyrosine nitration. However, it is known that the presence of one or more acidic residues and a relative paucity of cysteine, methionine and basic amino acids are required for tyrosine nitration to occur [22]. After searching the sequence of IRS-1 we have identified one tyrosine residue that responds to these criteria, the tyrosine at position 895 in rat or 896 in humans. Further experiments will be needed to determine whether this key tyrosine is the target of ONOO^−^ nitration in skeletal muscle.

One report showed that increasing ONOO^−^ with a NO/O_2_- donor (SIN-1) inhibited insulin-stimulated glucose transport and IRS-1-associated PI3K activity in 3T3-L1 adipocytes [35]. This was associated with increased nitration of tyrosine residues within IRS-1. However, the physiological relevance of such data is not clear as very high concentrations of SIN-1 were used in this paper. Moreover, we found no evidence for iNOS-linked IR in cytokine-exposed adipocytes or adipose tissue of obese mice [36], [37]. Since iNOS derived ONOO^−^ formation is dependent upon O_2_^−^ availability, the discrepancy regarding the role of iNOS as a direct mediator of IR in muscle and fat cells could potentially be the result of contrasting tissue reactive oxygen species profiles. Indeed, protein carbonyl levels, a marker of cumulative oxidative stress, were recently reported to be increased in liver but not in adipose tissue of mice fed a high fat diet for 6 weeks [38]. If a similar difference in oxyradical accumulation exists between muscle and adipose tissue, it could perhaps explain the differential impact of iNOS induction on insulin signal transduction in these two tissues. On this note it is important to mention that skeletal muscle oxygen consumption in the resting state is approximately 10 fold greater than that of white adipose tissue, potentially leading to greater mitochondrial O_2_^−^ generation in this tissue [39]. Alternatively, in the presence of equivocal O_2_^−^ generation, differences in O_2_^−^ scavenging among the two tissues could also contribute to a discrepancy in ONOO^−^ formation. Future studies should thus examine the influence that factors modulating O_2_^−^ generation and scavenging have on the impact of iNOS in insulin target tissues.

In conclusion, the present results obtained from rats, mice and L6 myocytes in culture strongly suggest that ONOO^−^ is a key mediator of skeletal muscle IR following iNOS induction. This oxidant derivative inhibits insulin action via tyrosine nitration of IRS-1 which impedes IRS-1 tyrosine phosphorylation and its associated PI3K activity. These findings provide new insights into the mechanism linking iNOS to skeletal muscle IR in inflammatory settings. Our work also presents nitrosative modification of insulin signaling proteins as a novel therapeutic target for improving muscle IR following LPS exposure during endotoxin shock or obesity-linked metabolic endotoxemia.

## Materials and Methods

### Ethics statement

This study was approved by the Animal Care and Handling Committee of Laval University (authorization number 08-032 and 00-041).

### Materials

Interferon-γ and TNF-α were purchased from Research Diagnostics Inc., (Flanders, NJ), and RD systems (Minneapolis, MN), respectively. 1400 W and polyclonal nitrotyrosine antibody were from Biomol Research Laboratories Inc. All cell culture solutions and supplements were purchased from Life Technologies, Inc. except for fetal bovine serum, which was purchased from Wisent (St-Bruno, QC, Canada). Reagents for SDS-polyacrylamide gel electrophoresis and immunoblotting were from Bio-Rad. ECL and [2-^3^H]Deoxyglucose were from PerkinElmer Life Sciences. [γ-^32^P]ATP, protein A- and G-Sepharose, anti-mouse or anti-rabbit immunoglobulin G conjugated to horseradish peroxidase were purchased from Amersham Pharmacia Biotech (Baie d'Urfé, QC, Canada). Polyclonal antibodies against IRS-1 (raised against 20 C-terminal amino acids (C-20)), were obtained from Santa Cruz Biotechnology (Santa Cruz, CA). The antibody IRS-1 for the western blot was from Cell Signaling (Beverly, MA). Polyclonal iNOS antibody was purchased from Upstate Biotechnology and eNOS and nNOS antibodies were from transduction (San Jose, CA). Monoclonal antibody against phosphotyrosine was obtained from Upstate Biotechnology (Lake Placid, NY). Human insulin was obtained from Eli Lilly (Toronto, ON, Canada). L-α -phosphatidylinositol was from Avanti Polar Lipids (Alabaster, AL). Oxalate-treated TLC silica gel H plates were obtained from Analtech (Newark, DE). All other chemicals were from Sigma (St.Louis, MO).

### Cell culture

L6 myoblasts were grown in α-MEM (10% FBS) containing 1% antibiotic/antimycotic (100 000 units/ml penicillin, 100 mg/ml streptomycin, 0.25 mg/ml amphotericin) and differentiated into myotubes in α-MEM (2% FBS) as previously described [11]. iNOS was induced by exposing cells to TNF-α (10 ng/ml), IFN-γ (200 U/ml) and LPS (10 µg/ml) for 24 hours. The incubation medium was kept to assess nitrite accumulation determined spectrophotometrically as described previously [11].

### Animals

Male Sprague-Dawley rats (200–250 g) purchased from Charles River (Montréal, Canada) were used in the aminoguanidine studies. Rats were pre-treated with a single intraperitoneal injection of aminoguanidine or saline (vehicle) 30 min prior to LPS (20 mg/kg, BW) or saline treatment. Animals were sacrificed 6 h later, and muscles tissues were removed and stored at −80°C until further processing. iNOS−/− mice (C57BL/6J crossbred), originally provided by C. Nathan and J.S. Mudgett (Cornell University) have been described previously [12]. The mice were kept under specific pathogen-free conditions at the Animal Facility of Laval University hospital research center and were given free access to a standard low-fat diet (Purina Rodent Chow, Madison, Wisconsin). For ex-vivo skeletal muscle glucose uptake experiments mice were sacrificed 6 h after LPS treatment (20 mg/kg).

### Hyperinsulinemic-euglycemic clamp procedure

Wild-type and iNOS−/− were randomly assigned to either saline or LPS (20 mg/kg) treatment groups. Five days prior to the experiment, mice were anesthetized and catheters inserted into the left common carotid artery and the right jugular vein for blood sampling and infusions, respectively. The free catheter ends were tunneled under the skin, externalized at the neck, and sealed. Mice were fasted for 5 h before the clamp procedure. The intravenous infusion catheters were connected to a swivel 1 h prior to the infusion, and the mice were unrestrained and not handled thereafter to minimize stress. Two and a half hours after LPS administration (*t* = 150 min), the hyperinsulinemic-euglycemic clamp (HIEC) was initiated according to previously described methodology [26], [40]. At the end of the clamp, mice were anesthetized, cardiac punctures were performed and hindlimb muscles were excised, freeze-clamped, and stored at −80°C until further analyses. Peripheral glucose uptake was determined using Mari's non–steady-state equations [41].

### Glucose uptake determinations

Glucose transport in isolated soleus muscles was measured using 2-[^3^H]2-deoxy-D-glucose as previously described [9]. 2-Deoxyglucose uptake in L6 myocytes was determined as previously described [11]. In brief, cells were incubated for 8 min in HEPES-buffered saline containing 10 µM unlabeled 2-deoxyglucose and 10 µM D-2-deoxy-[^3^H]glucose (0.5 µCi/ml). The reaction was terminated by washing three times with ice-cold 0.9% NaCl (w/v). Cell-associated radioactivity was determined by lysing the cells with 0.05N NaOH, followed by liquid scintillation counting.

### Measurement of Plasma NOx

Nitrite and nitrate (NOx) levels in plasma were measured by fluorometry. Briefly, blood was collected in tubes containing EDTA and centrifuged for 10 min at 3200× *g* to obtain plasma, which was then centrifuged at 5000× *g* (4°C) overnight in an Ultrafree®-Microcentrifuge 10,000 NMWL filter unit. Nitrate was reduced to nitrite using nitrate reductase and the NADPH regenerating system. The fluorescence was measured using 360 nm excitation and 450 nm emission.

### Tyrosine phosphorylation of the insulin receptor and IRS

Cell or tissue lysates (500 µg of protein) were immunoprecipitated with 2 µg of anti-phosphotyrosine (4G10) coupled to protein A-Sepharose overnight at 4°C. The immune complex was washed three times in PBS (pH 7.4) containing 1% NP-40 and 2 mmol/l Na_3_VO_4_ , resuspended in Laemmli buffer, and boiled for 5 min. Proteins were resolved on SDS-PAGE (6% gel) and processed for western blot analysis.

### PI 3-Kinase Activity

Cell or tissue lysates (500 µg) were immunoprecipitated with 2 µg of anti-IRS-1 coupled to protein A-Sepharose overnight at 4°C. PI 3-kinase activity was determined as previously described [42].

### IRS-1 nitrotyrosine measurement

Cell and tissue lysates (500 µg of protein) were immunoprecipitated with either 2 µg of IRS-1 antibody or 2 µg of anti-nitrotyrosine antibody coupled to protein G-Sepharose overnight at 4°C. The immune complex was washed three times in PBS (pH 7.4) containing 1% NP-40 and 2 mmol/l Na_3_VO_4_, resuspended in Laemmli buffer, and boiled for 5 min. Proteins were resolved on SDS-PAGE (7.5% gel) and processed for western blot analysis.

### Data Analysis

All data are presented as means ± SEM. The effects of the treatments were compared by an ANOVA analysis followed by Fisher's post hoc test. Differences were considered to be statistically significant at *P*<0.05.
